# Ethnic Differences in Serum Levels of microRNAs Potentially Regulating Alcohol Dehydrogenase 1B and Aldehyde Dehydrogenase 2

**DOI:** 10.3390/jcm10163678

**Published:** 2021-08-19

**Authors:** Ichiro Wakabayashi, Harald Sourij, Yoko Sotoda, Takashi Daimon, Klaus Groschner, Peter P. Rainer

**Affiliations:** 1 Department of Environmental and Preventive Medicine, Hyogo College of Medicine, Nishinomiya 663-8501, Japan; 2 Division of Endocrinology and Diabetology, Medical University of Graz, 8036 Graz, Austria; ha.sourij@medunigraz.at; 3 Department of Cardiovascular Surgery, Yamagata Saisei Hospital, Yamagata 990-8545, Japan; sotoda@ameria.org; 4 Department of Biostatistics, Hyogo College of Medicine, Nishinomiya 663-8501, Japan; daimon@hyo-med.ac.jp; 5 Gottfried Schatz Research Center for Cell Signaling, Metabolism and Aging, Medical University of Graz, 8036 Graz, Austria; klaus.groschner@medunigraz.at; 6 BioTechMed Graz, 8010 Graz, Austria; peter.rainer@medunigraz.at; 7 Division of Cardiology, Medical University of Graz, 8036 Graz, Austria

**Keywords:** alcohol dehydrogenase (ADH), aldehyde dehydrogenase (ALDH), bioinformatics, ethnic difference, microRNA

## Abstract

Ethnic difference is known in genetic polymorphisms of aldehyde dehydrogenase 2 (ALDH2) and alcohol dehydrogenase 1B (ADH1B), which cause Asian flushing by blood vessel dilation due to accumulation of acetaldehyde. We investigated ethnic differences in microRNAs (miRNAs) related to ALDH2 and ADH1B. miRNA levels in serum were totally analyzed by using miRNA oligo chip arrays and compared in Austrian and Japanese middle-aged men. There were no ALDH2- and ADH1B-related miRNAs that had previously been reported in humans and that showed significantly different serum levels between Austrian and Japanese men. With the use of miRNA prediction tools, we identified four and five miRNAs that were predicted to target ALDH2 and ADH1B, respectively, and they had expression levels high enough for comparison. Among the ADH1B-related miRNAs, miR-150-3p, -3127-5p and -4314 were significantly higher and miR-3151-5p was significantly lower in Austrian compared with Japanese men, while no significant difference was found for miR-449b-3p. miR-150-3p and miR-4314 showed relatively high fold changes (1.5 or higher). The levels of ALDH2-related miRNAs (miR-30d-5p, -6127, -6130 and -6133) were not significantly different between the countries. miR-150-3p and miR-4314 are candidates of miRNAs that may be involved in the ethnic difference in sensitivity to alcohol through modifying the expression of ADH1B.

## 1. Introduction

Aldehyde dehydrogenase 2 (ALDH2) is a major aldehyde-detoxifying enzyme, and its genetic polymorphism has been shown to cause flushing by dilation of blood vessels in East Asians [1] due to the accumulation of acetaldehyde after drinking alcohol. Facial flushing as a primary manifestation following alcohol ingestion is more frequent in East Asians (47~85%) than in Caucasians (3~29%) [2]. Other alcohol-induced acute symptoms in flushers are nausea, headaches and rapid heartbeat. Altered sensitivity to alcohol by polymorphism of *ALDH2* (rs671) is associated with the promotion of various diseases, including cancer [3] and cardiovascular disease [4]. ALDH2 contributes to the elimination of not only exogenous alcohol but also endogenous aldehydes, including lipid peroxidation products such as 4-hydroxynonenal, acrolein, malondialdehyde and crotonaldehyde. DNA damage induced by these endogenous aldehydes is considered to trigger various pathophysiological processes, including carcinogenesis, atherosclerosis, inflammation and neurodegeneration [5]. Another major alcohol-metabolizing enzyme is alcohol dehydrogenase 1B (ADH1B), which is related to the risk for alcohol dependence in Caucasians [6]. Individuals with ADH1B*1*1 (low activity type) are less sensitive to alcohol and tend to drink more alcohol compared to individuals with ADH1B*2*2 (high activity type). Polymorphism of *ADH1B* (rs1229984) is also known to determine sensitivity to alcohol [6] and is involved in Asian flushing [7].

MicroRNAs (miRNAs) are a class of endogenous short non-coding RNAs that act as post-transcriptional silencers by affecting messenger RNA (mRNA) stability based on sequence complementarity between each miRNA and its targeted mRNA. Five miRNAs have so far been reported to regulate the expression of ALDH2 (Table 1). In rat brain tissue, cerebral ischemia/reperfusion injury was reported to generate an increase in miR-193 expression, a decrease in ALDH2 expression and elevated levels of aldehydes, including malondialdehyde and 4-hydroxynonenal. In PC-12 cells originating from a rat pheochromocytoma, a miR-193 mimic inhibited ALDH expression and increased the levels of malondialdehyde and 4-hydroxynonenal, while a miR-193 inhibitor augmented ALDH expression and decreased the levels of these aldehydes [8]. Thus, miR-193 was shown to increase aldehyde levels by suppressing the expression of ALDH2 in rat neural tissue. The expression of ALDH2 was reportedly downregulated by miR-224 in bovine mammary epithelial cells [9], by miR-28 in mouse cardiomyocytes [10] and by miR-378a-5p in rat cardiomyocytes [11]. Using a bioinformatics approach, miR-34a was shown to downregulate ALDH2 in HepG2 cells, a human hepatocellular carcinoma cell line [12]. Collectively, these findings suggest that miRNAs play an important role in the regulation of ALDH2 expression in several species. On the other hand, there are only two reports on regulation of alcohol dehydrogenase (ADH) by miRNA: in human hepatoma cells, miR-1301-3p suppressed expression of ADH6 [13], while miR-148a-3p promoted ADH4 expression [14]. To the best of our knowledge, there have been no reports on miRNAs that regulate expression of ADH1B.

Here, we set out to investigate the possibility that differences in miRNAs regulating ALDH2 contribute to the phenomenon of Asian flushing. In this concise study, serum levels of the aforementioned five miRNAs, which have been reported to affect ALDH2 expression, were compared in Japanese and Austrian men as representatives of East Asians and Caucasians, respectively. Moreover, using bioinformatics prediction tools on miRNAs targeting, we searched for potential ALDH2- and ADH1B-related miRNAs, of which the serum levels were also compared between the two ethnicities.

## 2. Subjects and Methods

### 2.1. Subjects

Subjects were healthy Austrian (*n* = 20) and Japanese (*n* = 20) males who were not receiving any medications and were nonsmokers (age (mean with standard deviation): Austrians, 49.9 ± 6.3 years; Japanese, 48.7 ± 6.4 years). All of the Austrian subjects were Caucasians, and the Japanese subjects were originally from Japan. Subject characteristics (means with standard deviations) were as follows [15]: Height (180.3 ± 5.7 vs. 173.2 ± 4.5 cm), body weight (83.7 ± 9.1 kg vs. 70.0 ± 10.6 kg) and body mass index (25.7 ± 1.9 kg/m^2^ vs. 23.3 ± 3.1 kg/m^2^) were significantly higher in Austrian subjects than in Japanese subjects; systolic blood pressure (123.3 ± 10.1 mmHg vs. 123.4 ± 11.8 mmHg) and diastolic blood pressure (80.4 ± 5.3 mmHg vs. 79.5 ± 11.3 mmHg) were comparable between them; fasting sugar (78.8 ± 9.8 mg/dl vs. 97.7 ± 7.7 mg) and HDL cholesterol (47.3 ± 14.8 mg/dl vs. 61.6 ± 15.8 mg/dl) were significantly lower in Austrian than in Japanese subjects, while LDL cholesterol (145.7 ± 30.4 mg/dl vs. 124.3 ± 34.0 mg/dl) was significantly higher in Austrian than in Japanese men. The protocol of this study was approved by the Medical University of Graz Ethics Committee (27-166 ex 14/15) and the Hyogo College of Medicine Ethics Committee (No. 3036 in 2018). Written informed consent was provided by all of the participants.

### 2.2. Collection of Blood Sample

Blood was collected from each subject after overnight fasting. Serum was separated and kept frozen at −80 degrees until analysis of miRNAs.

### 2.3. RNA Extraction and miRNA Expression Profiling

RNA was extracted from a serum sample of each subject. Using an miRNA Oligo chip, 2565 miRNAs in serum were totally analyzed as described previously [15]. Briefly, half volumes of labelled RNAs were hybridized onto a 3D-Gene miRNA Oligo chip (Toray), which was designed to detect 2565 miRNA sequences, and the annotation and oligonucleotide sequences of the probes were conformed to the miRBase release 21, miRNA database (http://microrna.sanger.ac.uk/sequences/ (accessed on 29 October 2015)). After stringent washes, fluorescent signals were scanned with a 3D-Gene Scanner (Toray) and analyzed using 3D-Gene Extraction software (Toray). After normalization, each miRNA level with log2-transformation was compared between Austrian and Japanese participants. Fold change in the mean value of each miRNA intensity of Austrians vs. Japanese was calculated as the ratio of anti-log2 values of each mean of log2-transformed data in Austrian and Japanese men.

### 2.4. Bioinformatics Survey on miRNAs

Using miRNA target prediction databases, miRNAs related to ALDH2 and ADH1B were searched. We used two online programs, miRDB (http://mirdb.org (accessed on Mar 13, 2021)) and TargetScan (http://www.targetscan.org/vert_72/ (accessed on 13 March2021)). The criteria for selection of miRNAs were target score of 60 or higher in miRDB and context score of -0.3 or lower in TargetScan. Only the miRNAs that met both criteria were used for analysis.

### 2.5. Statistical Analysis

For each of the tested miRNAs, on the basis of the observed distribution of *p* values, we estimated the positive false discovery rate (*q* value), according to the method of Storey et al. [16]. All *q* values were two-sided, with statistical significance determined by a false discovery rate of less than 0.05.

## 3. Results

In our microarray analysis, 821 miRNAs showed serum levels that were high enough for statistical analysis. About 48% of these miRNAs were significantly different between Austrian and Japanese men. Among the miRNAs that showed a significant ethnic difference, 92 miRNAs (about 11% of total miRNAs with expression levels high enough for comparison) showed relatively high fold change (1.5 or higher). Thus, there is a considerable ethnic difference in miRNA expression.

Results of comparisons among the ALDH2-related miRNAs reported in previous papers [8,9,10,11,12] are shown in Table 1. Among the miRNAs that reportedly impact on ALDH2 expression, the level of miR-193a-5p was significantly higher in Japanese than in Austrian men, while there was no significant difference in miR-193b-5p levels. Serum levels of the other miRNAs that are considered to regulate ALDH2 such as miR-193a-3p, miR-193b-3p, miR-224 (miR-224-3p, miR-224-5p), miR-28 (miR-28-3p, miR-28-5p), miR-34a (miR-34a-3p, miR-34a-5p) and miR-378a-5p, were detected at levels too low for reasonable comparison between the ethnicities.

**Figure 1:**
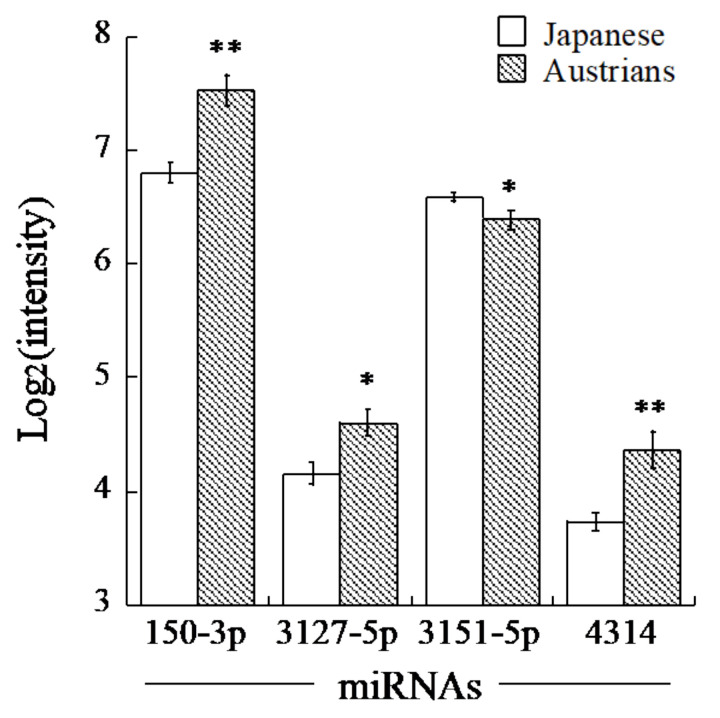
Comparison of miRNA levels in serum of Austrian and Japanese men. Shown are means of log2-transformed levels of each miRNA with their standard errors. Asterisks denote significant differences from the levels of Japanese (*, *q* < 0.01; **, *q* < 0.001).

By using the two miRNA target prediction databases, we selected eighteen miRNAs predicted to target ALDH2 and seven miRNAs for ADH1B (Table 2). Among these miRNAs, four miRNAs (miR-30d-5p, -6127, -6130 and -6133) for ALDH2 and five miRNAs (miR-150-3p, -3127-5p, -3151-5p, -4314 and -449b-3p) for ADH1B showed serum levels that were high enough for comparison. The mean levels of the ALDH2-related miRNAs, such as miR-30d-5p, 6127, -6130 and -6133, were not significantly different between Austrian and Japanese men. However, among the ADH1B-related miRNAs, our data revealed that miR-150-3p, -3127-5p and -4314 were significantly higher and miR-3151-5p was significantly lower in Austrian men than they were in Japanese men, while miR-449b-3p was not significantly different between the countries (Table 2, Figure 1). Only miR-150-3p and miR-4314 showed relatively high fold changes (1.5 or higher), while absolute fold changes of miR-3151-5p and miR-3127-5p were less than 1.5.

## 4. Discussion

This pilot study is the first study demonstrating ethnic differences in putatively alcohol-metabolizing enzyme-related miRNA expression in blood. Five miRNAs, which were predicted to target ADH1B and showed serum levels that were high enough for comparison, were extracted from the human databases. Among these, the mean levels of miR-150-3p, -3127-5p, -3151-5p and -4314 were significantly different between Austrian and Japanese men. Expression of miR-150-3p and miR-4314, (which were suggested to regulate expression of ADH1B according to miRNA target prediction databases) showed relatively high fold changes in the comparison (Austrian vs. Japanese men). Individuals with lower activity of ADH1B are prone to consume higher amounts of alcohol and show a higher incidence of alcohol use-associated disorders [6,7]. Therefore, negative regulation of ADH1B expression by miR-150-3p and miR-4314, resulting in slower metabolism of ethanol, is speculated to be stronger in Caucasians than in East Asians. This mechanism via ADH1B-related miRNAs might be involved in the ethnic difference in alcohol sensitivity in addition to polymorphism of the ADH1B gene. On the other hand, in the present study, we investigated eighteen miRNAs that have been suggested to regulate expression of ALDH2 by miRNA target prediction databases. Among them, only four miRNAs showed serum levels that were high enough for comparison, but these did not differ between Austrian and Japanese men. Thus, as far as we investigated, ALDH2-related miRNAs are not thought to be involved in ethnic difference in alcohol sensitivity, although further studies are needed to elucidate other miRNAs that regulate expression of ALDH2 and to determine whether miRNAs are involved in the ethnic difference in alcohol sensitivity, in addition to genetic polymorphism of ALDH2.

By searching for relevant miRNAs in the literature, we identified five miRNAs that have been reported to regulate expression of ALDH2 in different species [8,9,10,11,12]. Among them, only miR-193 levels in serum were high enough for comparison, and there was a significant difference in miR-193a-5p level between Austrian and Japanese men. The higher level of miR-193a-5p in Japanese men might cause a lower level of ALDH2 expression, resulting in more in vivo accumulation of aldehydes in Japanese men compared to Austrian men. However, experimental evidence from human studies is lacking. Although miR-193 has been reported to regulate ALDH2 expression in rat neuronal cells, miR-193 was not predicted to target ALDH2 in humans by our bioinformatics analysis. Therefore, it remains unclear whether miR-193 is involved in the regulation of ALDH2 expression in humans. Regarding miRNAs regulating ADH1B expression, to the best of our knowledge so far, no reports are available in the literature. In human hepatoma cells, expression of ADH6 is reportedly suppressed by miR-1301-3p [13], while expression of ADH4 was increased by miR-148a-3p [14]. However, serum levels of miR-1301-3p and miR-148a-3p in the subjects of this study were too low for a comparison between Austrian and Japanese men.

One limitation of this study is the relatively small size of the population comprised exclusively of male subjects. Thus, further extended studies using a larger number of subjects and female subjects are needed to confirm the findings of this study. The levels of miRNAs were analyzed by using a microarray in this study, and levels of some miRNAs related to ALDH2 and ADH1B were too low to measure correctly by this method. Therefore, analysis of miRNA expression level by using quantitative PCR is needed to confirm ethnic differences in the miRNAs that could not be evaluated due to too low expression in the blood in this study. There is, so far, only limited knowledge on the miRNAs linked to ALDH2 and ADH1B and, in particular, there is barely any information on these miRNAs from experimental studies using human materials and conservation between species. In addition, information on ALDH2 and ADH1B activities of the subjects was not available in this study.

## 5. Conclusions

Our pilot study suggests that miR-150-3p and miR-4314 may be involved in regulation of ADH1B expression. Ethnic differences in sensitivity to alcohol are mainly explained by mutants of *ALDH2* and *ADH1B* [5,6]. In addition to the well-known genetic polymorphism, there is a possibility that differences in the above two miRNAs are involved in the ethnic difference in alcohol sensitivity by affecting expression of ADH1B. We searched for ALDH2-related miRNAs from previous studies and bioinformatics databases and compared the candidate miRNAs between Austrian and Japanese men. Consequently, no ethnic difference was found in serum levels of these miRNAs, except for miR-193a-5p, which was reported to affect ALDH2 expression in rats (no reports of humans), and targeting of miR-193-5p to ALDH2 was not predicted in humans. The results of our pilot study strongly encourage further investigations to determine the roles of miRNAs as a regulator of the expression of alcohol-metabolizing enzymes in humans, as well as to elucidate their relevance for the pathophysiology of various alcohol-related diseases.

## Figures and Tables

**Table 1:** ALDH2-related miRNAs and comparison of their serum levels in Austrian and Japanese men.

miRNA	Regulation *	Material	Species	Reference	Comparison of Serum Level
miR-193	down	PC-12 cell, brain tissue	rat	Mao et al., 2017	*q* = 0.003 (miR-193a-5p) ** q = 0.100 (miR-193b-5p) n.d. (miR-193a-3p, miR-193b-3p)
miR-224	down	mammary epithelial cell	bovine	Shen et al., 2017	n.d. (miR-224-3p, miR-224-5p)
miR-28	down	cardiomyocyte	mouse	Li et al., 2015	n.d. (miR-28-3p, miR-28-5p)
miR-34a	down	HepG2 cell	human	Cheng et al., 2010	n.d. (miR-34a-3p, miR-34a-5p)
miR-378a-5p	down	cardiomyocyte	rat	Wang et al., 2017	n.d.

n.d., not determined due to low levels for comparison. *, “down” means suppression of ALDH2 expression by each miRNA in previous reports. **, the level was significantly lower in Austrian than in Japanese men (fold change: −1.29).

**Table 2:** Comparison of ALDH2- and ADH1B-related miRNA levels in serum of Austrian and Japanese men.

miRNA	Target Score	Context Score	Fold Change *	*q* Value
	(miRDB)	(TargetScan)		
*ALDH2*				
30a-5p	78	−0.39	n.d.	n.d.
30b-5p	78	−0.40	n.d.	n.d.
30c-5p	78	−0.40	n.d.	n.d.
30d-5p	78	−0.39	1.21	0.111
30e-5p	78	−0.41	n.d.	n.d.
3942-3p	90	−0.39	n.d.	n.d.
3689a-5p	65	−0.30	n.d.	n.d.
3689e-5p	65 **	−0.30	n.d.	n.d.
4510	66	−0.41	n.d.	n.d.
4753−3p	80	−0.34	n.d.	n.d.
4793-5p	75	−0.59	n.d.	n.d.
6127	66	−0.41	−1.04	0.272
6129	66	−0.41	n.d.	n.d.
6130	66	−0.43	−1.11	0.197
6133	66	−0.43	−1.01	0.412
6809-3p	94	−0.37	n.d.	n.d.
6854-3p	73	−0.47	n.d.	n.d.
8065	80	−0.60	n.d.	n.d.
*ADH1B*				
135b-3p	68	−0.41	n.d.	n.d.
150-3p	72	−0.35	1.65	<0.001
3127-5p	71	−0.32	1.36	<0.005
3151-5p	85	−0.42	−1.16	<0.003
3943	88	−0.34	n.d.	n.d.
4314	93	−0.52	1.55	<0.001
449b-3p	60	−0.34	−1.02	0.278

*, Fold change in each mean miRNA intensity of Austrian vs. Japanese men. **, miR-3689e in miRDB. n.d., not determined due to low levels for comparison.

## Data Availability

The datasets used and/or analyzed during the current study are available from the corresponding author on reasonable request.
